# Dynamic Mass Balance Modeling for Chemical Distribution Over Time in *In Vitro* Systems With Repeated Dosing

**DOI:** 10.3389/ftox.2022.911128

**Published:** 2022-08-22

**Authors:** Sherri Bloch, Jon A. Arnot, Nynke I. Kramer, James M. Armitage, Marc-André Verner

**Affiliations:** ^1^ Department of Occupational and Environmental Health, School of Public Health, Université de Montréal, Montreal, QC, Canada; ^2^ Centre de Recherche en Santé Publique, Université de Montréal et CIUSSS du Centre-Sud-de-l'Île-de-Montréal, Montreal, QC, Canada; ^3^ Department of Physical and Environmental Sciences, University of Toronto Scarborough, Scarborough, ON, Canada; ^4^ ARC Arnot Consulting and Research, Inc., Toronto, ON, Canada; ^5^ Division of Toxicology, Wageningen University, Wageningen, Netherlands; ^6^ AES Armitage Environmental Sciences, Inc., Ottawa, ON, Canada

**Keywords:** IVIVE, mass balance model, repeat dosing, dynamic model, facilitated transport

## Abstract

As toxicologists and risk assessors move away from animal testing and more toward using *in vitro* models and biological modeling, it is necessary to produce tools to quantify the chemical distribution within the *in vitro* environment prior to extrapolating *in vitro* concentrations to human equivalent doses. Although models predicting chemical distribution *in vitro* have been developed, very little has been done for repeated dosing scenarios, which are common in prolonged experiments where the medium needs to be refreshed. Failure to account for repeated dosing may lead to inaccurate estimations of exposure and introduce bias into subsequent *in vitro* to *in vivo* extrapolations. Our objectives were to develop a dynamic mass balance model for repeated dosing in *in vitro* systems; to evaluate model accuracy against experimental data; and to perform illustrative simulations to assess the impact of repeated doses on predicted cellular concentrations. A novel dynamic *in vitro* partitioning mass balance model (IV-MBM DP v1.0) was created based on the well-established fugacity approach. We parameterized and applied the dynamic mass balance model to single dose and repeat dosing scenarios, and evaluated the predicted medium and cellular concentrations against available empirical data. We also simulated repeated dosing scenarios for organic chemicals with a range of partitioning properties and compared the *in vitro* distributions over time. In single dose scenarios, for which only medium concentrations were available, simulated concentrations predicted measured concentrations with coefficients of determination (*R* ^2^) of 0.85–0.89, mean absolute error within a factor of two and model bias of nearly one. Repeat dose scenario simulations displayed model bias <2 within the cell lysate, and ∼1.5-3 in the medium. The concordance between simulated and available experimental data supports the predictive capacity of the IV-MBM DP v1.0 tool, but further evaluation as empirical data becomes available is warranted, especially for cellular concentrations.

## 1 Introduction

Currently, there are over 85,000 potentially toxic chemicals on the market, with more entering every year (Krimsky, 2017). However, health hazard information is lacking for a significant majority of commercial chemicals. Whole animal models are considered the traditional “gold standard” to investigate both deleterious endpoints and determine points of departure for risk assessment and regulatory decision-making. Unfortunately, the use of animal models comes with multiple limitations. *In vivo* animal testing requires a large number of laboratory animals to assess a single chemical and is a heavy strain on financial- and time-related resources, as well as a cause of ethical concern. Providing necessary safety assessments for all these chemicals within an acceptable time frame is impractical, leaving many chemicals data poor.

Furthermore, the scientific community has long been aware of the flaws in the predictive power and wide range of uncertainties associated with animal models, which even lead to removing certain drugs from the market (Blaauboer, 2010; Hartung, 2013). The influential report from the National Research Council (2007) recommended using *in vitro* data based on human-derived cells and organoids as initial models for chemical safety assessments. In addition, Europe has already banned the use of whole animal models for cosmetic testing, and the US EPA announced in 2019 that it aims to stop conducting and funding all mammal-based experiments by 2035 (Regulation, 2016; Grimm, 2019). Interest in the field of *in silico* predictive tools has increased to bypass the use of animal models. However, there are still obstacles to overcome on the way to raising confidence in *in silico* models for regulatory decision-making purposes.

In the interest of moving toward regulatory decisions based on *in vitro* data arising from human-derived cells and organoids, it is necessary to hone and perfect models relating to chemical biokinetics of the *in vitro* environment (Blaauboer, 2010; Bell et al., 2018; Zhang et al., 2018). Researchers commonly report a dose (concentration)-response relationship between the toxic effect and the nominal concentration (i.e., initial medium concentration), which may not correctly describe the effective concentration eliciting the biological response. The description of the kinetic behavior of the chemical within the *in vitro* system relates to the compound’s interaction with tissue/cells and the medium, binding to plastic and proteins, and volatilization (Blaauboer, 2010; Groothuis et al., 2015; Proença et al., 2021). Another factor to consider in the kinetics of the system is facilitated transport. It has been shown that through the use of proteins in media such as albumin and other dissolved organics, diffusive mass transfer of hydrophobic contaminants across unstirred aqueous boundary layers can be increased (Kramer et al., 2007; Mayer et al., 2007; Mayer et al., 2005; ter Laak et al., 2009a; ter Laak et al., 2009b). Due to facilitated transport, the chemical under investigation may therefore be transported into the cell at a faster rate in relation to the concentration of protein in the medium. Facilitated transport can also influence the kinetics of exchange across other interfaces (e.g., air-water). Furthermore, it may be important to take into account additional chemical mass which may be added to the system when medium is refreshed during longer-lasting experiments. Any miscalculation of the point of departure (PoD) at the level of chemical biokinetics in the *in vitro* system will translate into error when undergoing *in vitro* to *in vivo* extrapolation for human equivalent dose estimation.

To calculate the biologically effective dose in *in vitro* systems, mass balance models relying on empirical data and various quantitative structure-property relationships can be used. Proença et al. (2021) have grouped the available mass balance models into two types: static and kinetic (Proença et al., 2021). The static models are defined as models utilizing partition coefficients to calculate the compound concentrations in all phases present in the system at chemical equilibrium. Such models include the mass balance models by Gulden and Seibert (Gülden and Seibert, 2003), the Kramer model (Kramer, 2010), and the Fischer model (Fischer et al., 2017). Another static mass balance model is the IV-MBM EQP tool (Armitage et al., 2014), which has been applied in multiple studies (Casey et al., 2018; Hatherell et al., 2020; Zhang et al., 2020). Recently, the IV-MBM tool was updated and evaluated using available empirical data (Armitage et al., 2021). While all these models are very useful, they are limited in the sense that they effectively assume the *in vitro* system immediately achieves equilibrium, while it may take several hours or longer. However, it is possible that a response may be recorded prior to equilibrium, and the intracellular concentration at equilibrium predicted by the mass balance model has not yet been reached. Kinetic models are defined as utilizing differential equations parameterized with rate constants to simulate the distribution of chemical over time within the *in vitro* system. One of the earliest known kinetic models is the one developed by Zaldivar and colleagues (Zaldívar et al., 2010; Zaldivar Comenges et al., 2011). Another available kinetic mass balance model is by Fischer et al. (2020). While all these kinetic mass balance models predict medium and intracellular concentration over time, there is a general lack of model evaluation in relation to the growing number and diversity of chemicals that have been tested in *in vitro* systems. To increase the use of *in vitro* data for regulatory decision-making, current available *in silico* methods relating to the determination of a relevant dose metric need to be examined.

Here, we aimed to develop a novel dynamic partitioning mass balance model (IV-MBM DP v1.0) for *in vitro* repeated dosing experiments that accounts for volatilization, exchange within the medium, and facilitated transport. We also aimed to evaluate model accuracy against available experimental data, and to conduct illustrative simulations to assess the impact of repeated doses on predicted cellular concentrations for organic chemicals spanning a range of partitioning properties.

## 2 Methodology

### 2.1 Chemical and System Property Inputs

**FIGURE 1:**
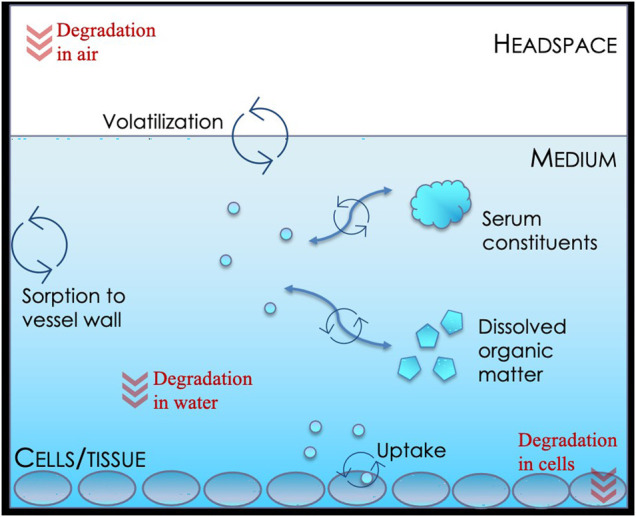
Schematic representation of the chemical biokinetics *in vitro* environment as described by the IV-MBM DP v1.0 model.

The IV-MBM DP v1.0 model (Figure 1) is implemented in a Microsoft Excel file and programmed using the Visual Basics for Applications (VBA) language. The user is required to define a set of physicochemical property inputs within the model, and is also able to enter degradation half-lives (HL) in air, medium and cells in hours (h) if available. In the absence of degradation rate estimates, the model code automatically assigns an arbitrarily long half-life such that the overall mass balance is not influenced by the rate. Quantitative structure-property relationships (QSPR) within the model are used to calculate the membrane-water, serum albumin-water, plastic-water partitioning coefficients, and the Setschenow constant (salting out constant); however, these estimated properties can be overridden by user entries. Similarly, the cell-water partition coefficient is calculated based on the proximate composition of the cell (i.e., lipid, protein, and water content) or defined by the user (Armitage et al., 2014). For more detail, please refer to the Supplementary Material.

There is an input sheet for system property inputs entitled “Well plate characteristics” that provides a selection of commonly used well plates with default dimensions listed or allows the user to input the dimensional parameters of the well plate. Next, the “Input system properties” sheet allows the user to define the *in vitro* system. For more detailed information, please see the Supplementary Material.

### 2.2 Intermedia Exchange, Degradation/Biotransformation, and Advective Transport

Intermedia exchange, degradation, and biotransformation, as well as advective transport are calculated using the well-established fugacity approach (Mackay, 2001) which takes into account information on flow rates and sorption capacities of the various phases. Intermedia exchange occurs between air and water, cells and water, and the vessel (plastic) and water. Degradation is calculated for the chemical in the headspace, the medium, and in the cells as a function of their respective reaction rate constants [*k* = ln (2)/HL, h^−1^], volumes, and sorption capacities. For all chemicals and simulations, degradation was assumed to be negligible in all phases and assigned an arbitrary half-life of 1e+10 h. For further explanation regarding calculations for degradation, please refer to Supplementary Material Section S1.3 of the Supplementary Material. Advective transport, i.e., the transport out of the headspace, is calculated as a function of volume of the headspace, the sorption capacity of air, and the rate constant describing exchange with air above the well plate. This rate constant is based on the residence time specified by the user. For more details, please see the Supplementary Material.

### 2.3 Facilitated Transport

The approach to estimate facilitated transport described by Kramer et al. (2007) was implemented in the IV-MBM DP v1.0 model as documented in the Supplementary Material. In brief, facilitated transport of the chemical across the unstirred aqueous boundary layer is a function of the concentration of serum albumin and other dissolved organics in solution, the corresponding partition coefficients and assumptions regarding the diffusivity of the bound and unbound form. Default parameter values included in the current implementation of the model can be overridden by the user. Note that facilitated transport is assumed to occur across all unstirred boundary layers and therefore influences the kinetics of cell-water exchange, air-water exchange and vessel (plastic)-water exchange.

As documented in the Supplementary Material, the facilitated transport factor (FTF) for bovine serum is calculated as:

$$FTF=1+L\;*\;\frac{D_{B}}{D_{U,i}}\;*\;K_{SaW}\;*\;C_{SaW}$$

where L is the lability factor (range = 0 to 1, default = 1), D_B_ is the diffusivity coefficient of the bound form in the aqueous phase (m^2^/h), D_U,i_ is the diffusivity coefficient of the unbound form of a given chemical in the aqueous phase (m^2^/h), K_SaW_ is the bovine serum albumin partition coefficient (L/L) and C_SaW_ is the concentration of serum albumin in the medium (L/L).

### 2.4 Repeat Dosing Exposure Scenarios

The IV MBM DP v1.0 model includes a worksheet titled “Input Exposure Scenario” which allows the user to input the test duration, the days on which medium is refreshed, the fraction of medium refreshed, and the nominal concentration of chemical in the new medium. For more detail, please refer to the Supplementary Material.

### 2.5 Model Parameterization and Evaluation: Single Dose Scenarios

The IV-MBM DP v1.0 model was parameterized and applied to simulate the chemicals and single dose scenarios described in Tanneberger et al. (2013) and Dupraz et al. (2019) The observations being compared to are the ratios of measured medium concentration at the end of the 24 or 96 h exposure period (C24 or C96) to the initial medium concentration (C0) (i.e., C24/C0 or C96/C0). Cellular concentrations were not measured in these studies.

The chemicals simulated for the Tanneberger et al. (2013) study (*n* = 27) and the Dupraz et al. (2019) study (*n* = 13) along with the required property data and initial nominal doses are compiled in the Supplementary Material. The simulated chemicals cover a wide range of hydrophobicity and volatility [e.g., the octanol-water partition coefficient (log K_OW_) and the air-water partition coefficient (log K_AW_) estimates span more than ten orders of magnitude] and thus are expected to behave very differently in *in vitro* test systems. The Tanneberger et al. (2013) study was conducted using a fish gill cell line (RTgill-W1) whereas the Dupraz et al. (2019) study was conducted using marine microalgae (*Tisochrysis lutea* and *Skeletonema marinoi*). Parameter values for the well plate characteristics, medium characteristics and cell line characteristics are also summarized in the Supplementary Material.

### 2.6 Model Parameterization and Evaluation: Repeat Dose Scenario

#### 2.6.1 Case Study 1: Data on Amiodarone

Experimental data was obtained from Pomponio et al. (2015). In the article, the team used mouse neurons isolated from frontal cortex tissue. They subsequently built a 2D mouse model of neurons. In addition to the physico-chemical information retrieved from PubChem, relevant information retrieved from the methods section of the article includes the plate measurements (6-well plate), working volume of 3 ml, medium composition (Primary Growth Medium SingleQuots catalogue No. CC-04462 and Primary Neuron Basal Medium calalogue No. CC-3256 from Lonza Sales AG, Viviers, Belgium), and the 14-days exposure scenario (amiodarone treatment of 1.25 μM, whole medium changes daily). The medium was not supplemented with serum lipids or albumin. However, the concentration of other dissolved constituents was calculated as 6 g/L, composed of dextrose at a concentration of 5 g/L, an approximate concentration of 0.7 g/L of amino acids, and 0.3 g/L of L-glutamine.

In comparison to serum lipids and albumin, it is more uncertain to what extent the presence of sugars such as glucose and other substances in solution can interact with test compounds added to the system. To simulate the potential influence of these dissolved substances on the distribution of the chemical, they were treated as dissolved organic matter (DOM) and assigned a partition coefficient scaled to K_OW_ using a proportionality constant (i.e., K_DOM_ = p K_OW_). The proportionality constants for K_DOM_ are based on the study by Burkhard (2000), which estimated the proportionality constant of K_DOM_ (p) to be equal to 0.08, and have 95% confidence limits of a factor of 20 in either direction. In addition, we also conducted simulations using the proportionality constant of 0.35 for organic carbon from Seth et al. (1999). For the Pomponio et al. (2015) study, the results using various K_DOM_ proportionality constants are found in the Supplementary Material, and the results below reflect only the default proportionality constant of *p* = 0.08. The partitioning properties compiled for amiodarone are summarized in Table 1.

**TABLE 1:** Properties of case chemicals used in repeat dose scenarios. Data retrieved from Pubchem https://pubchem.ncbi.nlm.nih.gov/ and the UFZ LSER database https://www.ufz.de/.

	MW	MP	LogK_ow_	LogK_aw_	C_sat,w_
Amiodarone	645.3	156.0	7.57	−8.52	2.69E-03
BDE-47	485	82	6.81	−2.83	6.40E-02

MW is molecular weight (g/mol); MP is melting point (°C), and C_sat,w_ is the water solubility at 25°C (mg/L).

#### 2.6.2 Case Study 2: Data on BDE-47

Experimental data was obtained from Schreiber et al. (2010). To determine the neurological effects of PBDEs, primary human fetal neural progenitor cells (hNPCs) were cultivated in the form of neurospheres and exposed to BDE-47. Neurospheres were exposed to BDE-47 over a period of 7 days, with half the medium (with a chemical concentration of 1 μM) being refreshed every second day. Exposure occurs in the Lab-Tek II Chamber slide (Thermo Fisher Scientific), which has a flat, square-based format. Culture area is reported at 0.7 cm^2^/well, with a total well volume of 907 μl and a working volume of 500 μl.

The medium used in these experiments was a mixture of Dulbecco’s modified Eagle medium (DMEM) and Ham’s F12 (3:1) with no supplementation with additional serum. Fischer et al. (2017) determined the lipid and protein concentrations within the DMEM solution as approximately 0.2 ml/L and 0.75 (0.69–0.86) ml/L respectively. The bulk medium in the model simulations was therefore parameterized to match these volume fractions. Again, we simulated the potential influence of other dissolved organics on the distribution of the chemical following the same approach as described in Case study 1. The partitioning properties compiled for BDE-47 are summarized in Table 1.

### 2.7 Model Parameterization: Illustrative Repeat Dose Scenarios for Selected Case Study Chemicals

To simulate the time-variant chemical distribution of chemicals displaying different physical and chemical properties, a group of chemicals with varying volatility and hydrophobicity were selected. For the “input chemical data” sheet, the chemical’s respective minimally required information were compiled and entered: the molecular weight (MW), the melting point (MP), the octanol-water coefficient (logK_OW_), the air-water coefficient (logK_AW_), and the water solubility at 25°C (C_SAT,W_). The input exposure scenario for all chemicals detailed on the “Input Exposure Scenario” sheet was for a duration of 7 days with an initial nominal medium concentration of 1 μM and half of the medium being replaced on days 2, 4, and 6 also with a nominal concentration of 1 μM. In the “well plate characteristics” sheet, the 96 well plate with a flat bottom was selected, with a bulk medium volume of 150 μl. Default serum characteristics of 24 g/L albumin and 1.9 g/L lipids were chosen, with only the volume fraction of serum varying from 0.02 to 0.20. To assess the potential importance of facilitated transport, the simulations assuming a serum volume fraction of 0.20 were repeated while disregarding facilitated transport (option available in input chemical data sheet).

For the study, we selected the following seven chemicals: acetone (very hydrophilic and volatile), dichloromethane (hydrophilic and volatile), 7-ethylbicyclooxazolidine (EBCO, hydrophilic and semi-volatile), D5 siloxane (very hydrophobic and semi-volatile), BDE-47 (very hydrophobic and non-volatile), disulfoton (moderately hydrophobic and non-volatile), and tetrachloroethylene (moderately hydrophobic and volatile).

Physicochemical properties of the chemicals were retrieved from PubChem (https://pubchem.ncbi.nlm.nih.gov/). In the case where the empirical value of a property was not available, such as logK_aw_ or C_sat,w_, values were estimated using the online UFZ LSER database (https://www.ufz.de/). The partitioning properties for the case study chemicals are summarized in Table 2.

**TABLE 2:** Chemicals used for illustrative model applications and their properties.

	MW	MP	LogK_ow_	LogK_aw_	C_sat,w_
Tetrachloroethylene	165.8	−22.3	3.40	0.36	206
Acetone	58.08	−96.55	−0.24	−2.58	1000000
BDE-47	485.79	82	6.81	−2.83	0.05
Siloxane D5	370.77	−38	8.06	2.87	0.017
Disulfoton	274.4	−25	4.02	−4.05	16.3
Dichloromethane	84.9	−95.1	1.25	−0.88	1.3E+04
7-ethylbicyclooxazolidine	143.19	15.43	0.4	−4.55	374600

MW is molecular weight (g/mol); MP is melting point (°C), and C_sat,w_ is the water solubility at 25°C (mg/L).

### 2.8 Metrics of Model Performance

#### 2.8.1 Single Dose Scenarios

Model bias (MB), mean absolute error (MAE) and the coefficient of determination (*R* ^2^) from linear regression models of measured vs. simulated levels were used to quantify the performance of the IV-MBM DP v1.0 model for the single dose scenarios. MB and MAE are calculated as shown below. Coefficients of determination (*R* ^2^) are calculated following the standard approach.

$$MB=\frac{\sum log_{10}\frac{P}{O}}{n}$$

$$MAE=\frac{\sum ABS\left(log_{10}\frac{P}{O}\right)}{n}$$

where P is a predicted value, O is the corresponding observed value, and *n* is the number of comparisons. As described above, the predicted and observed values are concentration ratios in the test medium between the end (24 or 96 h) and beginning of the exposure period (e.g., predicted vs. observed C24/C0). For ease of interpretation, MB and MAE are expressed as Factors of Agreement (FoA), i.e., 10^MB^ and 10^MAE^.

#### 2.8.2 Repeat Dose Scenarios

Model performance for repeat dosing aimed to compare experimentally derived results with those generated by the model for two case studies. For the interpretation of case study 1 (Pomponio et al., 2015), the MB expressed as FoA and the mean relative error were used.

$$Relative\;Error=100*\frac{\left|Estimated-Measured\right|}{Measured}$$

The mean relative error was used to measure the precision of the predicted data to the observed data. Case study 2 (Schreiber et al., 2010) was interpreted through comparison of the enrichment factor (EF) experimentally derived and the one calculated by the model, where

$$EF=\;\frac{Intracellular\;Concentration}{Nominal\;Concentration}$$

As mentioned above, we wanted to explore the sensitivity of the model results to the proportionality constant of the K_DOM_ (p) (Seth et al., 1999; Burkhard, 2000). We therefore generated EFs in accordance with each *p* and determined which *p* resulted in an EF closest to the empirical EF.

## 3 Results

### 3.1 Model Performance: Single Dose Scenarios

**FIGURE 2:**
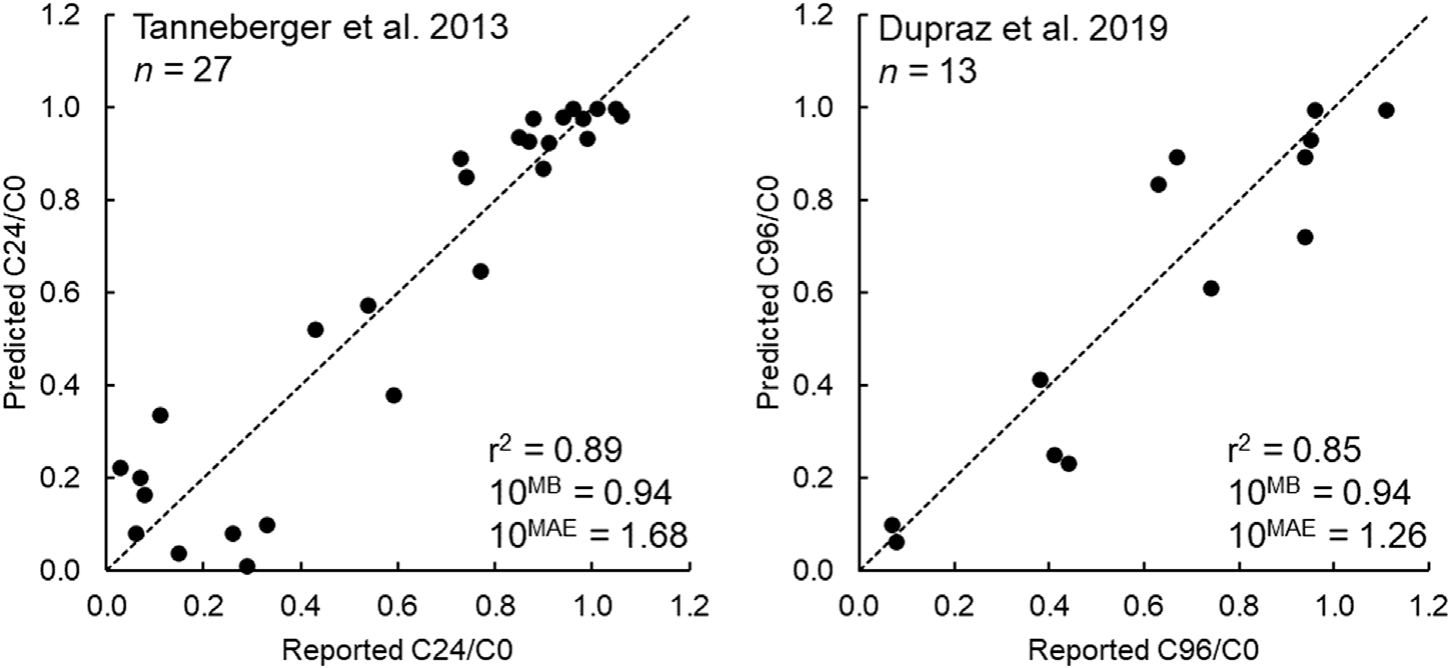
Model performance of the IV-MBM DP v1.0 model for the Tanneberger et al. (2013) (*n* = 27 chemicals) and Dupraz et al. (2019) (*n* = 13 chemicals) data sets.

The performance of the IV-MBM DP v1.0 model for the Tanneberger et al. (2013) and Dupraz et al. (2019) data sets are summarized in Figure 2. As shown, the coefficients of determination (*R* ^2^) were 0.89 and 0.85 for the two studies, and the mean absolute error (MAE) for medium concentrations corresponds to agreement within a factor of two for both data sets. The model bias (MB) is nearly one, indicating no systematic tendency to over- or underpredict the observed concentration ratios.

### 3.2 Model Performance: Repeat Dose Scenarios

#### 3.2.1 Case 1 on Amiodarone

**FIGURE 3:**
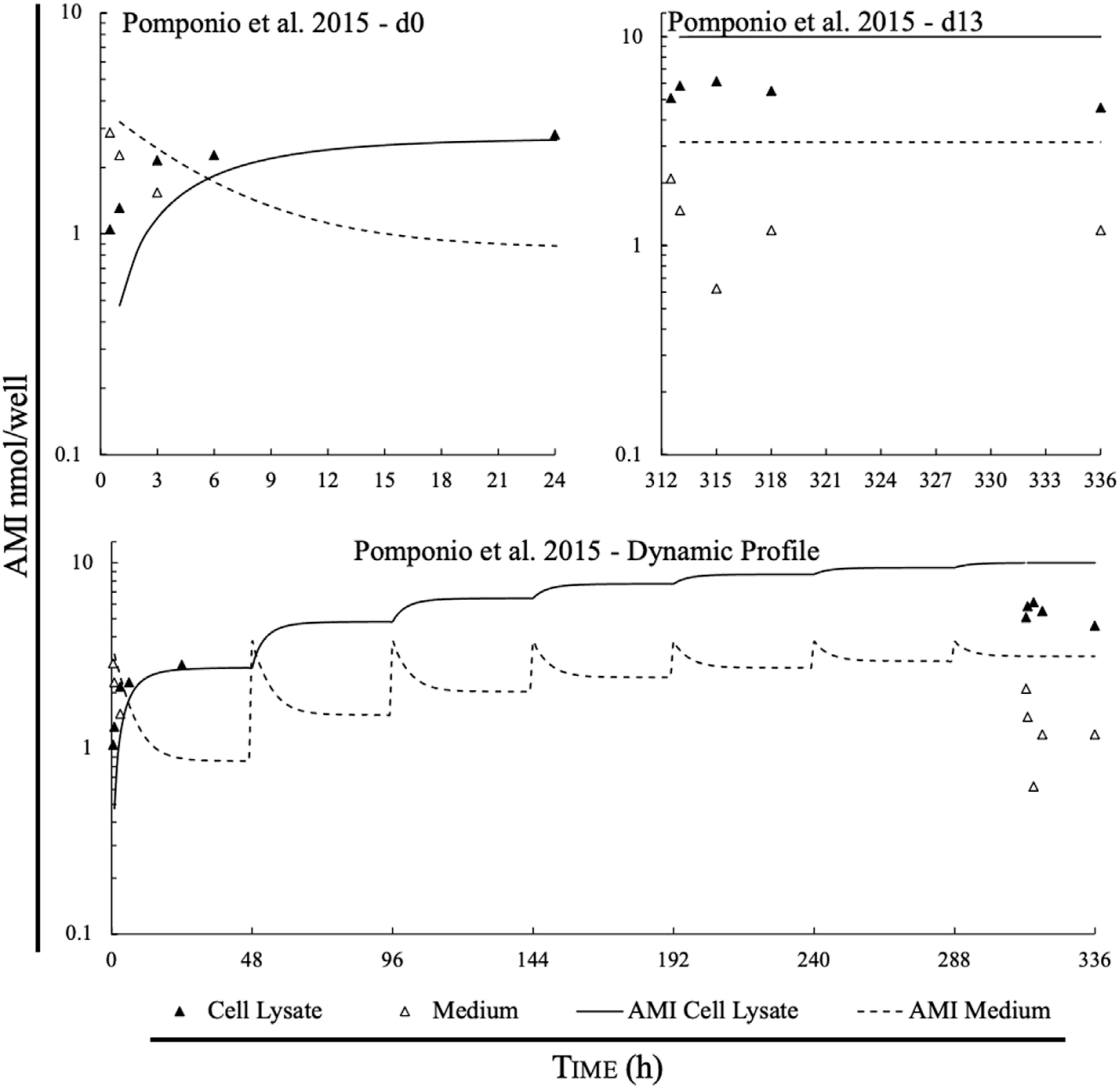
Comparison of measured data points and simulations of the IV-MBM DP v1.0 model for amiodarone (nmol/well) found in cell lysate and medium, given the experimental parameters described in the paper. Top left panel compares measured vs. predicted for day 0, top right panel depicts measured vs. predicted for day 13; bottom panel displays model performance of the predicted and the measured points of amiodarone mass throughout the experiment.

Figure 3 shows the observed data points in relation to predicted data calculated by the model. Predicted data points appear to have higher accuracy for both the medium and the cell lysate on day 0 compared to day 13. Furthermore, there seems to be higher accuracy for the prediction within the cell lysate than within the medium where an underprediction of chemical content is seen.

For the cell lysate (Figure 3, solid triangles), the model bias (MB) on day 0 and day 13 were −0.21 and 0.26, respectively. The MB within the lysate over the whole experiment was 0.027 due to overestimation of the concentration in the cell lysate on day 13. The mean relative error on days 0 and 13 were 72% and 83% respectively.

For the mass of amiodarone in the medium (Figure 3, open triangles), the MB on day 0 and day 13 were calculated at 0.18 and 0.47, respectively. The MB over the whole experiment within the cell lysate was 0.37. The mean relative error on day 0 and day 13 were 50% and 210%, respectively. However, due to measured data being below the LOD, there were only two points for day 0, and a total of six points in respect to the medium.

#### 3.2.2 Case 2 on BDE-47

**FIGURE 4:**
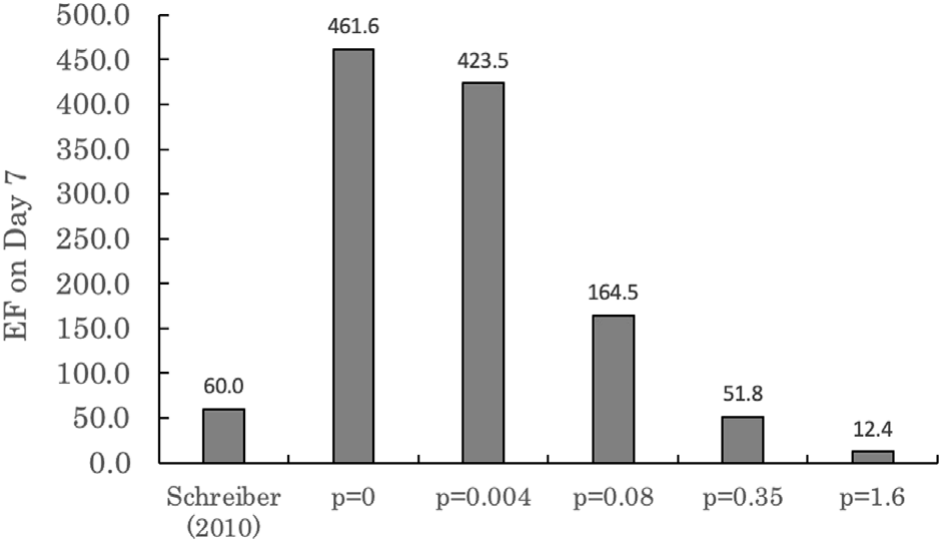
Enrichment factor (EF) on day 7 as calculated by the IV-MBM DP v1.0 model for Schreiber et al., 2010 with varying K_DOM_ proportionality constants (*p*). Where *p* = 0.004 and *p* = 1.6 are the lower and upper limits of the 95% confidence interval of *p* = 0.08 (Burkhard, 2000), and *p* = 0.35 is the proportionality constant to dissolved organic carbon (Seth et al., 1999).

Figure 4 presents the measured cellular enrichment factors (EF, concentration in cell divided by initial nominal medium concentration) and modeled EFs under different assumptions regarding the potential influence of medium constituents in addition to serum lipids and albumin. As seen in Figure 4, the enrichment factor (EF) observed by Schreiber et al. (2010) was approximately 60. The EFs calculated by the model on the final day were 482 (*p* = 0), 424 (*p* = 0.004), 165 (*p* = 0.08), 52 (*p* = 0.35), and 12 (*p* = 1.6). The proportionality constant described by Seth et al., (1999) yielded the EF prediction closest to the empirical value. However, these results do not provide conclusive evidence that sorption to additional medium constituents such as sugars and amino acids are responsible for the discrepancy under the scenario where these considerations are ignored (i.e., *p* = 0). As discussed below, there are other model uncertainties that must be considered when simulating the *in vitro* distribution of very hydrophobic chemicals.

### 3.3 Illustrative Model Applications: Repeat Dose Scenarios for Selected Case Study Chemicals

**FIGURE 5:**
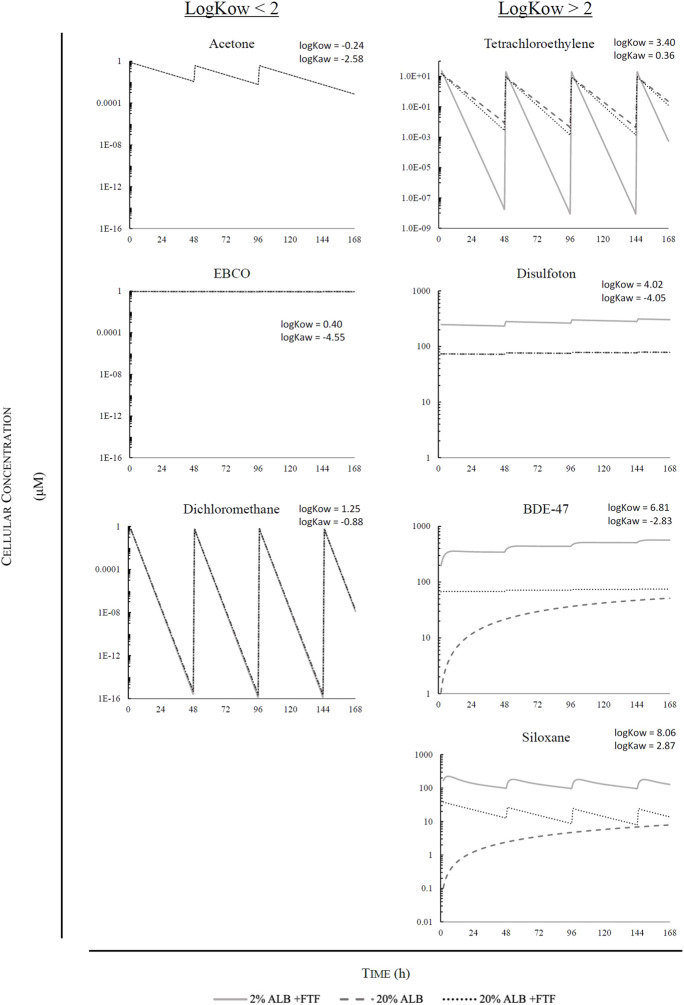
Graphic illustrations of simulations for chemicals with logK_OW_ < 2: Acetone, EBCO, and Dichloromethane; and illustrations of simulations for chemicals with logK_OW_ > 2: Tetrachloroethylene, Disulfoton, BDE-47, and Siloxane D5.

**FIGURE 6:**
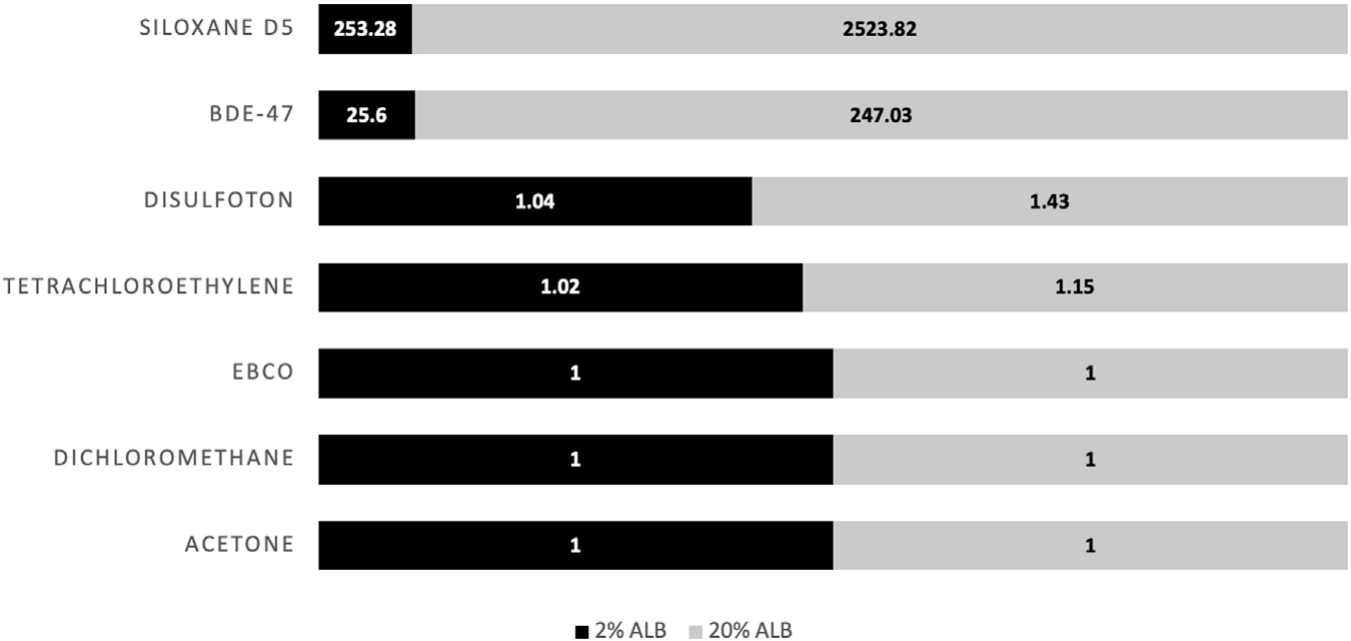
Facilitated transport factors (FTFs) for the different chemicals chosen as illustrative case chemicals in the presence of 2% (black) and 20% (grey) serum albumin.

Figure 5 displays results for predicted cellular concentration over time (μM) for the different case study chemicals assuming a serum volume fraction 0.02 and 0.20 with facilitated transport, and 0.20 while disregarding facilitated transport. Figure 6 displays the calculated facilitated transport factors (FTFs) for the different chemicals chosen as illustrative case chemicals in the presence of serum at volume fractions of 2% and 20%.

Of the different chemicals analyzed, acetone, dichloromethane, and EBCO were unaffected by serum content and facilitated transport, due to the low log K_ow_ of these chemicals. This is illustrated through the FTFs of approximately 1 in Figure 6.

Chemicals with high lipophilicity such as Siloxane D5 and BDE-47 are strongly affected by both serum content as well as facilitated transport. However, as BDE-47 is non-volatile and Siloxane D5 is volatile, the accumulated dose for BDE-47 is relatively stable when FTF is included, while the accumulated dose for Siloxane D5 is unstable and constantly increasing with FTF. As seen in Figure 6, Siloxane D5 has calculated FTFs of 253 and 2,520 at a serum volume fractions of 0.02 and 0.20, respectively. Due to repeat dosing and serum volume fractions, there is a higher cellular concentration at 0.02 vs. 0.20 volume fraction when taking FTF into account (9.37× higher). Furthermore, an increase by a factor of 1.74 is calculated for a 0.20 volume fraction due to FTF. For BDE-47, the FTFs calculated at volume fractions of 0.02 and 0.20 were 26 and 247, respectively. Due to repeat dosing and serum volume fractions, there is a higher cellular concentration at 0.02 vs. 0.20 volume fraction when taking FTF into account (7.55× higher). In addition, an increase by a factor of 1.46 is calculated for 0.20 volume fraction due to FTF.

Disulfoton which is moderately hydrophobic and non-volatile is strongly influenced by serum content, though not as strongly by facilitated transport. FTF ratios for disulfoton calculated at 0.02 and 0.20 serum volume fractions were 1.04 and 1.43, respectively (Figure 6). Due to repeat dosing and serum volume fractions, there is a higher cellular concentration at 0.02 vs. 0.20 volume fractions when taking FTF into account (3.86× higher).

Finally, tetrachloroethylene (log K_AW_ = 0.36) rapidly volatilizes out of the test medium, thus rendering cellular concentration close to null until the next exposure, when the chemical again accumulates in the cells. Facilitated transport has a very low influence on this chemical. FTF ratios for tetrachloroethylene assuming serum volume fractions of 0.02 and 0.20, were 1.02 and 1.15, respectively (Figure 6). There is a lower cellular concentration at 0.02 vs. 0.20 serum volume fraction when taking FTF into account (a factor of 4.3E-3) because substantially more chemical is lost from the medium *via* volatilization over time assuming the lower serum volume fraction. This trend in cellular concentration versus serum volume fraction is opposite to what occurs for the more hydrophobic chemicals (BDE-47, D5 siloxane) included here. For those chemicals, the influence of serum volume fraction on bioavailability and uptake into the cells is more important than the influence of bioavailability on air-water exchange. Furthermore, there was a decrease by a factor of 0.55 for the cellular concentration calculated for 0.20 serum volume fraction due to FTF after repeat dosing.

## 4 Discussion

As the scientific community advances toward *in vitro-in vivo* extrapolation (IVIVE) to increase resource efficiency and reduce ethical concerns in the context of human health risk assessment, it is necessary to advance tools aimed at the estimation of the effective dose/concentration in single dose and repeated dosing *in vitro* experiments. The IV-MBM DP v1.0 dynamic partitioning mass balance model developed herein can simulate chemical distribution in the *in vitro* system for single and repeat dosing, while taking into account volatilization and facilitated transport. This is necessary for longer-running experiments where medium is refreshed, especially when the chemical of interest is added with the refresher medium.

### 4.1 Model Performance: Single Dose Scenarios

The results comparing measured and predicted medium concentrations are encouraging and demonstrate the utility of the IV-MBM DP v1.0 model to predict chemical concentrations in medium over time. The additional advantage of the IV-MBM DP v1.0 model over the IV-MBM EQP v2.0 version (Armitage et al., 2021) is that concentrations in all compartments over time are generated as output, providing deeper insight into the dynamics within the *in vitro* system over the time course of exposure. The dynamic partitioning (DP) version is more computationally intensive and much lower throughput however. Comparisons between model output generated by the equilibrium (EQP) and dynamic partitioning (DP) versions of the model would provide insight into when use of the DP version is warranted but are outside the scope of the current study.

Empirical *in vitro* disposition data including cellular concentrations as well as the amount of chemical sorbed to plastic are not widely available. For this reason, the experimental data used for evaluation was confined to medium concentrations, which limited our evaluation. As with the performance of the IV-MBM EQP model (Armitage et al., 2021), the accuracy of the partitioning data, estimated cell properties (e.g., mass, seeding density, and proximate composition) and estimated surface area of plastic available for sorption are key considerations. Given the dimensional information, the estimated surface areas are relatively well constrained; however, data on the proximate composition of the numerous cell lines used for *in vitro* toxicity testing are sparce, e.g., (Fischer et al., 2017; Henneberger et al., 2019; Henneberger et al., 2020). Generating such data is relatively straightforward and is considered a priority for future research in order to increase confidence in mass balance model applications.

### 4.2 Model Performance: Repeat Dose Scenario

#### 4.2.1 Simulations of Amiodarone

On the first day, the model showed a tendency to underestimate the concentration within the cell lysate and overestimate the concentration in the medium. However, although their MB was low, their mean relative errors were high, and displaying lowered precision. By the final day, the MB as well as the mean relative error increased for both the lysate and the medium. The tendency to overpredict product in the lysate and medium by the final day may be due to the underprediction of depletion of chemical in the *in vitro* environment. In the paper by Pomponio et al. (2015), metabolism of amiodarone by the isolated neurons into MDEA appeared to be insignificant, with no metabolism recorded on day 0 and a limited amount recorded on the final day. It may be possible that amiodarone is being transformed into an unmeasured metabolite, or perhaps it is being degraded in solution. There is an option within the model to calculate chemical degradation (input chemical data sheet); however, degradation half-lives are required for this option, and this information is not readily available and must be experimentally derived for the specific medium used in the experiment. Another possibility is the adjustment of the proportionality constants for dissolved organic matter. This constant would also need to be determined by the experimenting laboratory.

#### 4.2.2 Simulations of BDE-47

To cover a range of proportionality constants to octanol for K_DOM_, a variety of *p* values based on published literature (Seth et al., 1999; Burkhard, 2000) were assumed for these simulations. The proportionality constant with the result closest to the empirical value was *p* = 0.35 (Seth et al., 1999). However, confidently predicting the EF is difficult due to multiple reasons and these results do not prove that these assumptions regarding sorption to dissolved organics are accurate. One unknown factor is the growth of the neurospheres over the course of the experiment. Without knowing the exact volume of the neurospheres, it is more difficult to accurately predict the intracellular concentration of the chemical. A second unknown factor is the actual lipid content of the hNPCs cells provided by Lonza Verviers SPRL. This factor is very important given the lipophilicity of BDE-47 (log K_OW_ = 6.81). Moreover, Schreiber et al. (2010) reported that at the end of the experiment, 91% of the BDE-47 present within the *in vitro* environment was adsorbed to the plastic vessel. In contrast, a similar experiment by Mundy et al., (2004) with BDE-47 determined a fraction bound to plastic closer to the one produced by the IV-MBM DP v1.0 model. Our model produced a fraction bound to plastic of 0.4% (*p* = 1.6) to 11.9% (*p* = 0), while the empirical fraction bound to plastic determined by the investigation of Mundy et al. (2004) produced a fraction associated with the tissue culture plate of 20% to 40%. Adjusting the default estimated plastic-water partition coefficient to better match the reported distribution from Schreiber et al. (2010) yields a cellular EF within a factor of two of the observed value. However, whether the difference between the empirical EF and predicted EF is due to the bias in plastic-water partitioning, the presence of other dissolved organics such as sugars and amino acids in the medium, or any of a multitude of undetermined factors is unknown.

### 4.3 Illustrative Model Applications: Repeat Dose Scenarios for Selected Case Study Chemicals

These chemicals chosen for the illustrative applications reflect the ability of the model to predict the biokinetics of chemicals with varying degrees of volatility and hydrophobicity. In addition, the model was used to determine the effect of facilitated transport in the presence of different concentrations of albumin in solution (Figure 6). Overall, the model shows the effects of the physio-chemical properties of the chemicals, as well as the FTF on the predicted intracellular concentration. The model shows that in the case of hydrophilic chemicals, whether they are volatile, semi-volatile, and non-volatile (i.e., acetone, dichloromethane, and EBCO), there is a freely dissolved volume fraction approaching 100% regardless of serum volume fraction assumptions, leaving sorption to serum lipids and albumin insignificant. In the case of tetrachloroethyene, which is classified as a low to moderately hydrophobic and volatile substance, a decreased serum volume fraction as well as FTF leads to a lower accumulated intracellular concentration due to the increased loss of chemical into the headspace. In the case of a non-volatile, low to moderately hydrophobic substance such as disulfoton, the FTF has a limited influence on uptake kinetics while the volume fraction of albumin has a greater effect. The very hydrophobic and non-volatile BDE-47 is also strongly influenced by the albumin volume fraction. As the serum volume fraction increases, the system will reach a steady state more quickly due to facilitated transport. However, more of the chemical will be bound to serum lipids and albumin, resulting in a lower bioavailable fraction. If facilitated transport is ignored, the accumulation of BDE-47 in cells is much slower due to the very small fraction of the chemical that is directly available for diffusive uptake. In the case of siloxane D5 (log K_OW_ = 8.06, log K_AW_ = 2.87), facilitated transport also results in much quicker accumulation of the chemical by the cells. However, because facilitated transport is also assumed to occur at the air-water interface, volatilization of the chemical out of the test system is more rapid and results in declines in predicted cellular concentrations between the repeat doses. If facilitated transport is ignored, uptake into the cells and losses due to volatilization are both much slower, again because of the very small fraction of the chemical that is freely-dissolved and directly available for cell-water and air-water exchange. The patterns predicted here regarding the relationship between medium protein concentration and bioavailability are in agreement with those seen in the study by Fischer et al. (2018). Studying uptake kinetics in the *in vitro* environment through use of fluorophores, Fischer et al. (2018) determined that increasing fetal bovine serum (FBS) concentrations leads to increased chemical partitioning to lipids and proteins found in the medium, thus leading to decreased bioavailability.

The descending order of chemicals that appeared to be the most highly affected by serum in solution were siloxane D5, BDE-47, disulfoton, and tetrachloroethylene; for EBCO, dichloromethane, and acetone, predictions were minimally influenced by albumin content. In addition, it is important to note that the model demonstrates that although the dosing regime is exactly the same for all chemicals, the accumulated dose inside the cell at the end of the simulation is different for every substance and can vary by many orders of magnitude. Overall, the illustrative model applications clearly demonstrate the variation in concentration for different chemicals given identical nominal exposure scenarios and highlight the problematic use of nominal concentrations for both single and repeat dose scenarios.

### 4.4 Merits and Limitations

Current trends regarding data published by the *in vitro* toxicology community are usually in the form of a relationship between the studied toxic effect and the nominal medium concentrations. Such data may be misleading as the nominal concentration may not reflect the exposure experienced by the cells following chemical distribution within the *in vitro* environment. The IV-MBM DP v1.0 model presented herein can be used to estimate freely-dissolved and cellular concentrations corresponding to reported nominal medium concentrations in studies where these concentrations are not measured. The model can also be used in a reverse dosimetric fashion prior to an experiment to tailor the chemical concentration within the cell and the medium to the needs of the study. However, further efforts are required to increase confidence in the use of such tools. Empirical *in vitro* disposition data for chemicals in cells, medium and sorbed to plastic would help in the parametrization and evaluation of the model. Specifically, more robust evaluations of the model predictions for repeat dose scenarios require experiments where the chemical is tested at multiple concentrations and concentrations are measured at multiple time points in cells and medium. Multiple concentrations are necessary for the identification of saturation processes and the contribution of uptake and excretion, as seen by the difference in kinetic profiles for saturation in Wilmes et al. (2013). Furthermore, small but necessary details such as lipid, protein, and dissolved organic matter content within the medium used for the experiment need to be measured and/or reported. Another limitation involves the lack of information regarding cell cultures used for *in vitro* testing. Though mass and concentrations of lipid, protein, and dissolved organic matter are necessary for more reliable predictions, information regarding these constituents is not always publicly available (OECD, 2018).

## 5 Conclusion

Through this study, we have shown the importance of the components of the *in vitro* environment (e.g., albumin, lipids, and plastic) when determining the effective dose-response relationship for both short- and long-term dosing scenarios. We believe that a better understanding of the potential influence of serum constituents other than lipids and albumin on *in vitro* disposition would also be useful for future model applications. This is particularly true for the various additives and supplements used in repeat dose studies. As much of this is either confidential or not reported, it may require regulatory authorities and peer-reviewed journals to demand greater transparency.

## Data Availability

The original contributions presented in the study are included in the article/Supplementary Material, further inquiries can be directed to the corresponding author. The IV-MBM DP v1.0 model is also available at https://arnotresearch.com/models/.
