# Supplementary material for: Dynamic Mass Balance Modeling for Chemical Distribution Over Time in In Vitro Systems With Repeated Dosing
Source: Front Toxicol. 2022 Aug 22;4:911128. doi: 10.3389/ftox.2022.911128 (PMC9441784; doi:10.3389/ftox.2022.911128)
Supplement: Supplementary file 2 [file DataSheet1.docx]

***Supplementary Material***

# Model Description

The In Vitro Mass Balance Model “Dynamic Partitioning” (IV-MBM DP v1.0) is a fugacity-based (Mackay 1979, MacKay 2004) tool for simulating the disposition of neutral organic chemicals in in vitro test systems over time. It was developed to complement the updated equilibrium partitioning based model (IV-MBM EQP v2.0) described in a separate publication (Armitage, Sangion et al. 2021).

The EQP and DP models are based on the Armitage et al. 2014 model (Armitage, Wania et al. 2014) but with significant updates for the EQP version and updates and changes to the calculation methods for the DP version. Whereas the updated IV-MBM EQP model is suitable for high-throughput calculations of single dose scenarios for multiple chemicals/assays, the IV-MBM DP model was developed to address some of the limitations inherent to the EQP approach (e.g., treatment of volatilization, inability to consider degradation/biotransformation, cell growth) and also to allow the user to explicitly simulate repeat dosing scenarios.

Both the IV-MBM EQP v2.0 and IV-MBM DP v1.0 are currently implemented as Microsoft Excel/VBA applications. Simulations using the IV-MBM DP tool can only be conducted on a chemical-by-chemical basis thereby increasing the computational and user effort required to generate results for multiple chemicals/testing scenarios in comparison to the EQP version.

## Required and Optional Chemical Property Inputs

The minimal set of physical-chemical properties required to run the IV-MBM DP tool are as follows:

1. Molecular weight (MW, g/mol)
2. Melting point (MP, ^o^C)
3. Octanol-water partition coefficient at 25 ^o^C (log K_OW_)
4. Dimensionless air-water partition coefficient at 25 ^o^C (log K_AW_)
5. Water solubility at 25 ^o^C (C_SAT,W_, mg/L)

The user is also able to enter degradation half-lives in air, medium and cells (HL, h) which are automatically converted to degradation rate constants (k = ln2/HL, 1/h) and subsequently used in the model calculations. In the absence of user-defined degradation half-lives, the model code automatically assigns a negligible rate constant (1e-20 per h) so that degradation has a negligible influence on the overall mass balance (i.e., the chemical is assumed to be very persistent)

Optional inputs for physical-chemical properties that the user can enter include the following:

1. Entropy of fusion (dS_M_, J/mol/K)
2. Internal energy of phase change (octanol-water) (dU_OW_, J/mol)
3. Membrane-water partition coefficient 25 ^o^C (log K_MW_)
4. Internal energy of phase change (membrane-water) (dU_MW_, J/mol)
5. Internal energy of phase change (air-water) (dU_AW_, J/mol)
6. Plastic-water partition coefficient at 25 ^o^C (log K_PLASTIC_ in units of m^3^/m^2^)
7. Cell-water partition coefficient (log K_CW_)
8. Serum albumin-water partition coefficient (log K_SaW_)
9. Setschenow constant (K_SALT_)

The entropy of fusion is used along with melting point to adjust the properties of chemicals present as solids at test system temperature to the sub-cooled liquid properties required for the chemical activity calculations generated by the model. See Armitage et al. (Armitage, Wania et al. 2014) and references therein for further explanation of the chemical activity concept and its application for interpreting toxicity data.

Internal energies of phase change are used to adjust partitioning properties at 25 ^o^C to the test system temperature using the van’t Hoff equation (Schwarzenbach, Gschwend et al. 2003).

Membrane-water, serum albumin-water and plastic-water partitioning data entered by the user override the default calculations otherwise made to estimate these properties using various Quantitative Structure-Property Relationships (QSPRs) (e.g., (Endo, Escher et al. 2011, Endo and Goss 2011)). The cell-water partition coefficient (log K_CW_), if entered by the user, will override the composition-based estimation approach described in Armitage et al. (Armitage, Wania et al. 2014) and expanded upon in the updated EQP model (i.e., K_CW_ as a function of storage lipid, membrane lipid, bulk protein and water content and the corresponding partition coefficients).

With respect to plastic-water partitioning, two default QSPRs to estimate this property value are provided as options for the user to consider:

Option 1 (Kramer 2010)

$logK_{PLASTIC}=0.97\cdot logK_{OW}-6.94$

Option 2 (Fischer, Cirpka et al. 2018)

$logK_{PLASTIC}=0.56\cdot logK_{OW}-4.64$

Note that Option 2 is the regression published recently by Fischer et al. but converted to the units of m^3^/m^2^ required by the IV-MBM DP tool.

Finally, the Setschenow constant (K_SALT_) is used to adjust water solubility and partitioning properties to account for ‘salting out’ effects at the ionic strength of the medium (default = 0.15 M) (Schwarzenbach, Gschwend et al. 2003). In the absence of a user-entered value, the following QSPR is utilized (Ni and Yalkowsky 2003):

$$K_{SALT}=0.04\cdot logK_{OW}+0.114$$

Additional details on these input properties and options for estimating missing inputs are documented on the “Input Chemical Data” sheet of the IV-MBM DP tool.

## Required and Optional System Property Inputs

The user is first required to select or define the characteristics of the well plates to be simulated on the “Well Plate Characteristics” sheet of the IV-MBM DP tool. The following properties are included:

1. Well diameter (mm) and bottom area (mm^2^)
2. Total well volume (µL)
3. Working volume (i.e., volume of medium added to well) (µL)
4. Average cell yield/initial seeding density (# of cells/well)
5. Mass of an individual cell (ng)

Default parameter values are provided for 6, 12, 24, 48, 96, 384 and 1536 well plates. The user is able to override these defaults but only experienced users should proceed with such modifications. For example, the IV-MBM DP tool does not check to see if the entered values are plausible.

The user is then required to define other key characteristics of the test set-up on the “Input System Properties” sheet of the IV-MBM DP tool. The following properties are included:

1. Proximate composition of cells (storage lipid, membrane lipid and protein content where water is calculated as the remainder) and density
2. Doubling time (i.e., time required for the volume of cells present to double – linear, default = blank i.e., no growth)
3. Temperature and ionic strength of test medium
4. Serum and “other dissolved organic matter” volume fractions in medium
5. Characteristics of serum added (concentration of serum albumin and lipids in g/L)
6. Volume of headspace (µL) – recommended value = Total well volume – Working volume
7. Advective residence time in headspace (h)

The combination of serum volume fraction in medium (typical range = 0 – 0.20) and serum characteristics are used to estimate the volume of serum albumin and serum lipids in the test medium.

The advective residence time in the well headspace is set to an arbitrary value of 0.1 h to reflect the combination of advective and diffusive transport. For many chemicals of interest, the rate-limiting step for air-water exchange is diffusion across the unstirred boundary layer on the water-side anyway. To simulate a “sealed well”, the user should set the advective resident time to an arbitrarily large value (e.g., 100 000 h).

Additional details on these input properties and options for estimating missing inputs are documented on the “Input System Properties” sheet of the IV-MBM DP tool.

## Intermedia Exchange, Degradation/biotransformation and Advective Transport

Intermedia exchange, degradation/biotransformation and advective transport processes in fugacity-based models are described using “transport D-values”. D-values are calculated using information on flow or process rates (m^3^/h) and the sorption capacity of the phases of interest, termed Z-values in units of mol m^-3^ Pa (Mackay 2001).

Intermedia exchange processes considered in the IV-MBM DP tool include air-water exchange, vessel-water exchange and cell-water exchange. These exchange processes are modeled using the standard “two resistance” approach whereby the overall mass transfer coefficient (MTC) is calculated from, for example, the air-side and water-side MTCs in series.

*Air-water Exchange*

$$D^{AW}=\frac{1}{\frac{1}{({MTC}_{aw}\cdot A\cdot Z_{A})}+\frac{1}{({MTC}_{wa}\cdot A\cdot Z_{W}\cdot FTR)}}$$

where D^AW^ is the D-value for air-water exchange in both directions, MTC_aw_ is the mass transfer coefficient on the air-side of the boundary layer, A is the surface area of exchange between air and water (function of well plate characteristics), Z_A_ is the sorption capacity of air, MTC_wa_ is the mass transfer coefficient on the water-side of the boundary layer, Z_W_ is the sorption capacity of water and FTR is the Facilitated Transport Ratio introduced in the main text and calculated as detailed below.

Pure air is taken as the reference phase in the IV-MBM DP tool and its Z-value is defined as shown below:

$$Z_{A}=\frac{1}{R\cdot T}$$

where R is the Gas Law Constant (8.314 Pa m^3^ mol^-1^ K^-1^) and T is the test system temperature in Kelvin (K). Z-values for other phases are calculated using partition coefficients. The Z-value for water is shown below:

$$Z_{W}=\frac{Z_{A}}{K_{AW}}$$

The value of the air-side MTC_aw_ is assigned the standard value of 3.6 m/h for still conditions recommended by Mackay (Mackay 2001). The water-side MTC_wa_ is calculated from the diffusivity coefficient of the chemical in water (m^2^/h) divided by pathlength (m), assuming the boundary layer thickness on the water-side is 1/3^rd^ the height of the test medium. The diffusivity coefficient of the chemical in water is estimated as described in the Facilitated Transport section below (i.e., scaled to the diffusivity coefficient of carbon dioxide in water).

### Cell-water Exchange

$$D^{CW}=\frac{1}{\frac{1}{({MTC}_{cw}\cdot A\cdot Z_{C})}+\frac{1}{({MTC}_{wc}\cdot A\cdot Z_{W}\cdot FTR)}}$$

where Z_C_ is the sorption capacity of the cells and is calculated using the cell-water partition coefficient K_CW_) and A is the surface area of exchange between the cells and water.

$$Z_{C}=K_{CW}\cdot Z_{W}$$

K_CW_ is a user-entered value or calculated using the proximate composition of the cell and the corresponding partition coefficients. See previous section. The surface area of exchange between the cells and water is estimated by treating the cells as a single sphere covering the bottom of the well plate, with ¾ exposed to water and ¼ shielded from direct contact. This estimate is therefore a function of the well plate characteristics. The specific surface area of the cells (m^2^/kg) is provided as an output on the “Kinetic Information” sheet of the IV-MBM DP tool.

The cell-side MTC_cw_ is the membrane permeability (log P) of the chemical which is estimated the approach selected by the user on the “Input Chemical Data” sheet of the IV-MBM DP tool:

Option 1 (Trapp and Horobin 2005)

$logP=logK_{OW}-6.7$ in units of m s^-1^ (then converted m h^-1^)

Option 2 (Yazdanian, Glynn et al. 1998, Zaldivar, Mennecozzi et al. 2011)

$logP=-1.1711+0.98\cdot logK_{OW}-0.0011\cdot MW$ in units of cm h^-1^ (then converted to m h^-1^)

The water-side MTC_wc_ is given the same value as for air-water exchange (see above).

### Vessel-water Exchange

Sorption to plastic is treated as an adsorptive process in the IV-MBM DP tool and hence only water-side transport is explicitly considered.

The Z-value of plastic is calculated similarly as the Z-value of cells and the water-side MTC is given the same value as for air-water exchange (see above).

### Degradation/biotransformation

In headspace

$$D^{RA}=k_{R,A}{\cdot V}_{A}\cdot Z_{A}$$

In test medium

$$D^{RW}=k_{R,W}{\cdot V}_{W}\cdot Z_{W}$$

In cells

$$D^{CW}=k_{C,W}{\cdot V}_{C}\cdot Z_{C}$$

where k, V and Z are reaction rate constants, volumes and sorption capacities for each phase respectively. The reaction rate constants are assigned an arbitrary value of 1e-20 1/h in the absence of user-entered values or calculated from the user-entered half-lives (HL) following the standard conversion, i.e., k = ln2/HL if provided.

### Advective transport (out of headspace)

The transport D-value for this process is as follows:

$$D^{HA}=k_{ha}\cdot V_{A}\cdot Z_{A}$$

where k_ha_ is the rate constant for advective transport out of the headspace. This rate constant is calculated from the residence time (τ,h) entered by the user following the standard conversion, i.e., k = 1/τ

## Facilitated Transport (FTF)

Facilitated transport refers to the enhanced exchange kinetics between water and solids (e.g., SPME fibres, membranes, cell walls) or other boundary layers (e.g., air-water) observed in the presence of dissolved organic matter, bovine serum albumin and other dissolved solids (e.g., (Oomen, Mayer et al. 2000, Kramer, van Eijkeren et al. 2007, Mayer, Fernqvist et al. 2007, ter Laak, van Eijkeren et al. 2009)). The basic explanation for the enhanced exchange kinetics is that the presence of dissolved solids in the unstirred water boundary layer facilitates the total diffusive mass transfer by effectively increasing the apparent flux of unbound chemical. This will occur as long as desorption of bound chemical from solids in the unstirred boundary layer into the freely-dissolved phase is rapid i.e., not the rate-limiting step in the overall exchange kinetics. Facilitated transport is more evident i) in systems with greater concentrations of dissolved organic matter in the aqueous phase and ii) for more hydrophobic chemicals. Facilitated transport does not influence the “equilibrium position” of the chemical with respect to partitioning between the *freely-dissolved* aqueous phase and the other phase but simply shortens the time for equilibrium to be achieved. However, the presence of dissolved (and particulate) matter reduces the apparent “equilibrium position” between for e.g., a solid and the aqueous phase if the concentration in water is referenced to *total concentration*. This reduction in bioavailability due to the presence of particulate and dissolved matter has been observed in both toxicity and bioconcentration testing.

Facilitated transport is addressed in the IV-MBM DP model using a Facilitated Transport Factor (FTF) following Kramer et al. (Kramer, van Eijkeren et al. 2007). The FTF is a function of the concentration of dissolved solids in the culture medium (serum lipids, serum albumin, “other dissolved organic matter”, DOM), a lability factor and the ratios of the diffusivities of the bound and unbound chemical.

For bovine serum albumin, the FTF is calculated as shown below:

$FTF=1+L\cdot\frac{D_{B}}{D_{U.i}}\cdot K_{SaW}\cdot C_{SaW}$

where L is the lability factor (range = 0 to 1, default = 1), D_B_ is the diffusivity coefficient of the bound form in the aqueous phase (m^2^/h), D_U,i_ is the diffusivity coefficient of the unbound form of a given chemical in the aqueous phase (m^2^/h), K_SaW_ is the bovine serum albumin partition coefficient (L/L) and C_SaW_ is the concentration of serum albumin in the medium (L/L). The lability factor L can be reduced from the default value of 1 to approximate the potential impact of kinetic limitations in desorption from the bound state to the freely-dissolved (i.e., bioavailable) state.

D_B_ is the same for all chemicals whereas D_U,i_ varies with molecular weight of the chemical. The diffusivity of the unbound chemical in the aqueous phase is scaled to the estimated diffusivity of carbon dioxide (CO_2_) in water (Schwarzenbach, Gschwend et al. 2003) using molecular weight (MW), i.e.,

$D_{U,i}=D_{U,CO2}\cdot\left( \frac{{MW}_{i}}{{MW}_{CO2}} \right)^{-0.5}$

Where D_U,CO2_ is the diffusivity coefficient of carbon dioxide in water, MW_i_ is the molecular weight of the chemical of interest and MW_CO2_ is the molecular weight of carbon dioxide (44 g/mol).

$$D_{B}=D_{U,CO2}\cdot\left( \frac{66500}{{MW}_{CO2}} \right)^{-0.5}$$

where 66500 is the approximate molecular weight of serum albumin (66.5 kDa = 66500 g/mol).

Assumptions regarding the FTF are given on the “Input Chemical Data” sheet of the Excel/VBA implementation of the IV-MBM DP tool. The user is also presented with an option (check box) to “Disregard Facilitated Transport”. If selected, the FTF is set to 1 for all chemicals and test conditions.

## Repeat Dosing Exposure Scenarios

Single or repeat dosing scenarios are defined on the “Input Exposure Scenario” sheet of the Excel/VBA implementation of the IV-MBM DP tool.

The user is required to enter the test duration (d) and then the initial nominal medium concentration (µM). To simulate a repeat dosing scenario, the user then enters the time of the next dose, the fraction of medium replaced and the nominal concentration aimed for with the subsequent doses.

## Numerical Integration

The time-variant calculations are conducted using numerical integration using a time step (h) selected based on the log K_OW_ of the chemical (1e-4 to 1e-5 h). The fugacity (i.e., *f* = concentration / Z in units of Pa) of the medium compartment is initialized using the initial nominal medium concentration whereas the fugacities of all other compartments are set to zero.

Uptake and loss from each bulk compartment (headspace, medium, cells, vessel wall) are quantified over time using the standard ordinary differential equations implemented for “Level 4” (i.e., time-variant) fugacity-based models.

The fluxes for the exchange, degradation and advection processes are simulated over time as the product of fugacities and D-values (*f*·D in units of mol/h) and used to update the inventory in each compartment per time step. Cell growth is accounted for at the end of each iteration by converting the cell fugacity to mass (M = fZ/V), increasing the total cell volume, recalculating concentration and then fugacity.

The various calculated values described below are then output to the spreadsheet at designated times (hourly) over the simulated exposure period.

## Outputs Generated by IV-MBM DP

The following outputs are automatically generated and displayed by the Excel/VBA version of the IV-MBM DP tool.

1. Predicted concentrations in headspace air, bulk medium, sorbed to serum albumin, lipids and DOM, freely-dissolved, cells and cell membrane and amount sorbed to plastic (µmoles/m^2^) over time
2. Nominal Depletion Factors (DF) and Cell Enrichment Factors (EF) over time
3. Mass fractions associated with all compartments including precipitate over time
4. Masses associated with all compartments including precipitate over time, the cumulative amounts degraded in air, medium and cells over time, the cumulative amount advected out of the well headspace over time and the total mass balance over time
5. Chemical activity over time (see Armitage, Wania et al. 2014 and references therein for further explanation)
6. Kinetic information (e.g., permeabilities, mass transfer coefficients), estimate specific cell surface area and the predicted Facilitated Transport Factor (FTF)

These outputs can be found in the “Concentrations”, “Mass Fractions”, “Mass Balance”, “Chemical Activity” and “Kinetic Information” sheets of the IV-MBM DP tool.

# Additional details on model parameterization and application: Single dose scenarios

The key input parameters describing the test system parameters for the three single dose applications are summarized in Supplementary Tables 1 and 2.

**Supplementary Table 1.** Key Input parameters for the Tanneberger, Knöbel et al. (2013) simulations

| **Input Parameter** | **Selected Value** |
| --- | --- |
| *Well plate characteristics*  Well plate size  Well diameter  Growth area  Total well volume  Volume of medium added  *Medium characteristics*  Temperature  pH  Ionic strength  FBS volume fraction (%)  *Cell characteristics*  Cell seeding density  Mass per cell  Total mass of cells  Storage lipid content  Membrane lipid content  Protein content  Water content | 24  15.6 mm  190 mm^2^  3400 µL  2000 µL  19 ^o^C  7.4  0.15 M  0.00  300 000  0.525 ng  0.158 mg  0.01  0.04  0.055  0.895 |

**Supplementary Table 2.** Key Input parameters for the Dupraz, Stachowski-Haberkorn et al. (2019) simulations

| **Input Parameter** | **Selected Value** |
| --- | --- |
| *Well plate characteristics*  Well plate size  Well diameter  Growth area  Total well volume  Volume of medium added  *Medium characteristics*  Temperature  pH  Ionic strength  FBS volume fraction (%)  *Cell characteristics*  Cell seeding density  Mass per cell  Total mass of cells  Storage lipid content  Membrane lipid content  Protein content  Water content | 48  11 mm  95 mm^2^  1700 µL  1000 µL  19 ^o^C  7.4  0.15 M  0.00  20000  5.0 ng  0.1 mg  0.01  0.04  0.05  0.90 |

# Compositions of culture media used in the single and repeat dose experiments

## Single Dose Experiments

The experiments reported in Dupraz, Stachowski-Haberkorn et al. (2019) were conducted using f/2 and f/2-Si medium whereas the experiments reported in Tanneberger, Knöbel et al. (2013) were conducted using L15/ex medium. Details on these two media are summarized below

### f/2 medium

<http://web.biosci.utexas.edu/utex/Media%20PDF/f-2-medium.pdf>

**Supplementary Table 3. Composition of f/2 medium**

| **Component** | **Amount** | **Final concentration** |
| --- | --- | --- |
| NaNO_3_  NaH_2_PO_4_·H_2_O  Na_2_SiO_3_·9H_2_O  Trace Metals Solution  Vitamin B12  Biotin Vitamin Solution  Thiamine Vitamin Solution | 1 ml  1 ml  1 ml | 880 µM  36 µM  106 µM  1 mL/L  1 mL/L  1 mL/L  1 mL/L |

### L-15/ex

As explained in Schirmer, Chan et al. (1997) L-15/ex is a modified version of the L-15 medium formulation where all components except inorganic salts, galactose and pyruvate (pyruvic acid) are excluded. The excluded additives include various amino acids, vitamins and L-glutamine.

<https://www.sigmaaldrich.com/CA/en/technical-documents/technical-article/cell-culture-and-cell-culture-analysis/mammalian-cell-culture/l-15-media-formulation>

**Supplementary Table 4.** Composition of L-15/ex medium

| **Component** | **Final Concentration (g/L)**  **L1518** |
| --- | --- |
| *Inorganic salts*  Calcium chloride  Magnesium chloride  Magnesium sulfate (anhydrous)  Potassium chloride  Potassium phosphate Monobasic (anhydrous)  Sodium chloride  Sodium phosphate Dibasic (anhydrous) | 0.1396  0.0937  0.0977  0.4  0.06  8.0  0.19 |
| *Other*  D-galactose  Pyruvic acid | 0.90  0.55 |

## Repeat Dose Experiments

### Pomponio, Zurich et al. (2015)

Pomponio, Zurich et al. (2015) describes the medium as containing primary neuron basal medium (PNBM) and supplemented with the primary neuron growth medium (PNGM) SingleQuots Kit. Both products were acquired from Lonza Sales AG, Viviers, Belgium, catalogue No CC-3256 and CC-4462. The components of the solutions were proprietary, thus the concentrations below are approximate. Neither product contained lipid or albumin components. The addition of all components below lead to a total DOM of 5.975 g/L.

**Supplementary Table 5. Composition of medium used in repeat dose experiment by Pomponio, Zurich et al. (2015)**

| **Component** | **Final Concentration (g/L)**  **PNBM and PNGM** |
| --- | --- |
| Dextrose  Amino acid | 4.934  0.747 |
| *Other*  L-glutamine | 0.292 |


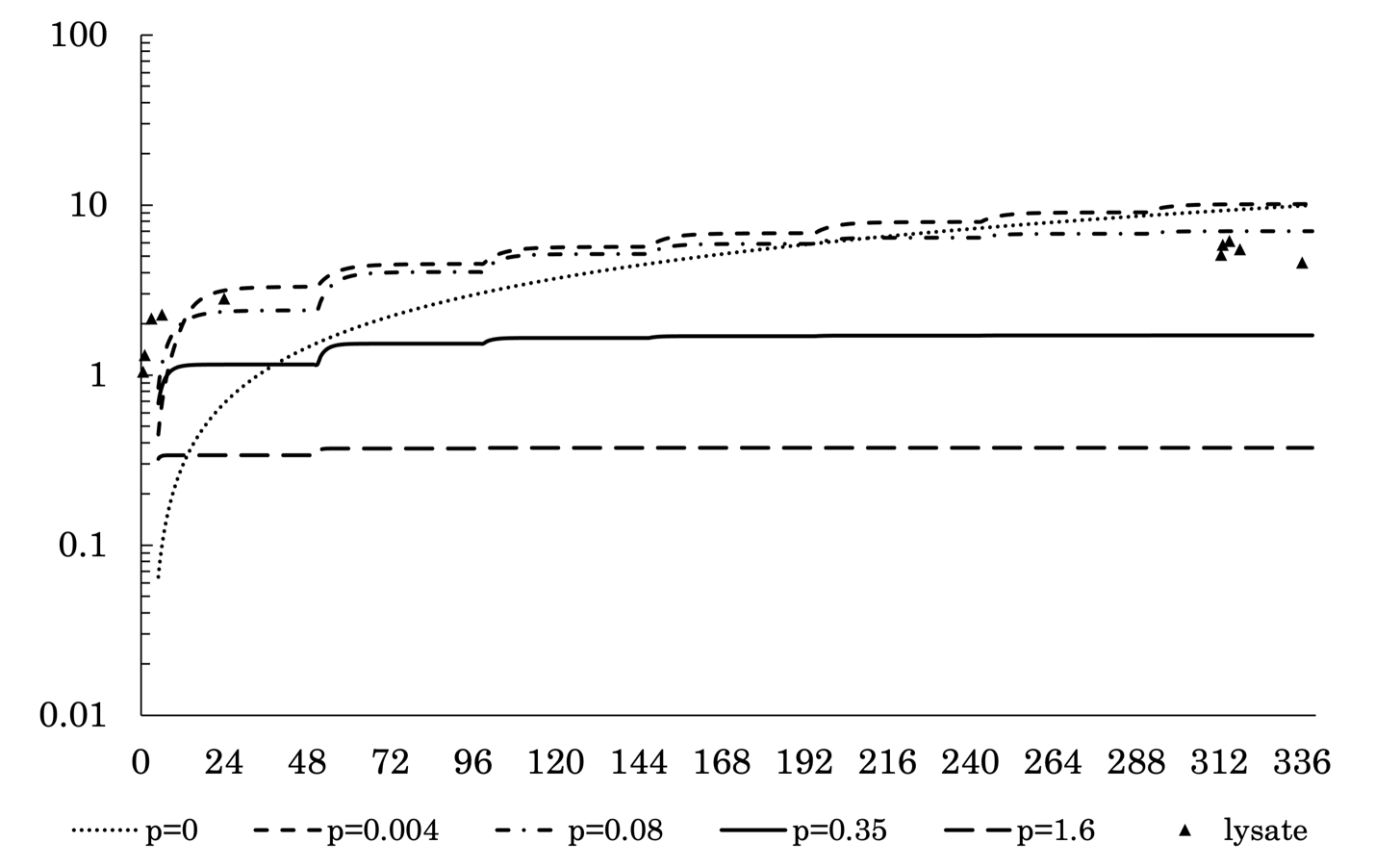


**Supplementary Figure 1.** Demonstrates the dynamics of the concentration within the cell lysate with varying proportionality constants (p) to K_DOM_ over time (hours) in relation to the measured data.

The graph demonstrating the predictions with varying p, in reference to Seth, Mackay et al. (1999) and Burkhard (2000). In this scenario, p=0.08 (Burkhard 2000) yields predictions closest to the measured data at both time points. The p=0 scenario (i.e., ignoring sugars and other dissolved organics) also generates predictions in good agreement with the empirical data at the end of the experiment but not near the beginning.

**Supplementary Table 6.** Relative error per data point for Pomponio, Zurich et al. (2015) data. This analysis represents the predictions resulting from default parametrizations found in main text.

| time (h) | lysate | medium |
| --- | --- | --- |
| 1 | 176.82% | 41.88% |
| 3 | 82.31% | 58.23% |
| 6 | 23.92% | n/a |
| 24 | 6.06% | n/a |
| 313 | 70.77% | 111.87% |
| 315 | 62.74% | 401.55% |
| 318 | 81.39% | 163.33% |
| 336 | 117.40% | 163.19% |

### Schreiber, Gassmann et al. (2010)

Fischer, Henneberger et al. (2017) reported the following protein and lipid contents for various culture media (Supplementary Table 7).

**Supplementary Table 7**. Protein and lipid contents reported for various culture media

| Medium | Protein content  (mL/L) | Lipid content (mL/L)  (gravimetric method) | Lipid content (mL/L)  (Sulpho-phospho-vanillin method) |
| --- | --- | --- | --- |
| DMEM Glutamax  Phenol Red DMEM  OptiMEM | 0.86  0.69  1.59 | 0.81  0.80  0.95 | 0.19  0.20  0.15 |

According to Fischer, Henneberger et al. (2017), "the sulfo-phosphovanillin method was found to be most suitable for the lipid determination of *in vitro* test media and cells due to the small amounts of lipids that rendered the gravimetric method unreliable.” The IV-MBM DP v1.0 model was therefore parameterized manually to approximate the protein (assumed to be albumin) and lipid contents of DMEM Glutamax and Phenol Red DMEM (0.75 ml/L albumin, 0.2 ml/L lipid).

# References

Armitage, J. M., A. Sangion, R. Parmar, A. B. Looky and J. A. Arnot (2021). "Update and Evaluation of a High-Throughput In Vitro Mass Balance Distribution Model: IV-MBM EQP v2.0." Toxics **9**(11).

Armitage, J. M., F. Wania and J. A. Arnot (2014). "Application of Mass Balance Models and the Chemical Activity Concept To Facilitate the Use of in Vitro Toxicity Data for Risk Assessment." Environmental Science & Technology **48**(16): 9770-9779.

Burkhard, L. P. (2000). "Estimating Dissolved Organic Carbon Partition Coefficients for Nonionic Organic Chemicals." Environmental Science & Technology **34**(22): 4663-4668.

Dupraz, V., S. Stachowski-Haberkorn, J. Wicquart, N. Tapie, H. Budzinski and F. Akcha (2019). "Demonstrating the need for chemical exposure characterisation in a microplate test system: toxicity screening of sixteen pesticides on two marine microalgae." Chemosphere **221**: 278-291.

Endo, S., B. I. Escher and K.-U. Goss (2011). "Capacities of Membrane Lipids to Accumulate Neutral Organic Chemicals." Environmental Science & Technology **45**(14): 5912-5921.

Endo, S. and K.-U. Goss (2011). "Serum Albumin Binding of Structurally Diverse Neutral Organic Compounds: Data and Models." Chemical Research in Toxicology **24**(12): 2293-2301.

Fischer, F. C., O. A. Cirpka, K.-U. Goss, L. Henneberger and B. I. Escher (2018). "Application of Experimental Polystyrene Partition Constants and Diffusion Coefficients to Predict the Sorption of Neutral Organic Chemicals to Multiwell Plates in in Vivo and in Vitro Bioassays." Environmental Science & Technology **52**(22): 13511-13522.

Fischer, F. C., L. Henneberger, M. König, K. Bittermann, L. Linden, K.-U. Goss and B. I. Escher (2017). "Modeling Exposure in the Tox21 in Vitro Bioassays." Chemical Research in Toxicology **30**(5): 1197-1208.

Kramer, N. I. (2010). Measuring, modeling, and increasing the free concentration of test chemicals in cell assays. PhD, Unversity of Utrecht.

Kramer, N. I., J. C. van Eijkeren and J. L. Hermens (2007). "Influence of albumin on sorption kinetics in solid-phase microextraction: consequences for chemical analyses and uptake processes." Anal Chem **79**(18): 6941-6948.

Mackay, D. (1979). "Finding fugacity feasible." Environmental Science & Technology **13**(10): 1218-1223.

Mackay, D. (2001). Multimedia Environmental Models - The Fugacity Approach, 2nd Edition. Boca Raton, FL, USA, CRC Press LLC.

MacKay, D. (2004). "Finding fugacity feasible, fruitful, and fun." Environmental Toxicology and Chemistry **23**(10): 2282-2289.

Mayer, P., M. M. Fernqvist, P. S. Christensen, U. Karlson and S. Trapp (2007). "Enhanced Diffusion of Polycyclic Aromatic Hydrocarbons in Artificial and Natural Aqueous Solutions." Environmental Science & Technology **41**(17): 6148-6155.

Ni, N. and S. H. Yalkowsky (2003). "Prediction of Setschenow constants." Int J Pharm **254**(2): 167-172.

Oomen, A. G., P. Mayer and J. Tolls (2000). "Nonequilibrium Solid-Phase Microextraction for Determination of the Freely Dissolved Concentration of Hydrophobic Organic Compounds:  Matrix Effects and Limitations." Analytical Chemistry **72**(13): 2802-2808.

Pomponio, G., M.-G. Zurich, L. Schultz, D. G. Weiss, L. Romanelli, A. Gramowski-Voss, E. Di Consiglio and E. Testai (2015). "Amiodarone biokinetics, the formation of its major oxidative metabolite and neurotoxicity after acute and repeated exposure of brain cell cultures." Toxicology in Vitro **30**(1, Part A): 192-202.

Schirmer, K., A. G. Chan, B. M. Greenberg, D. G. Dixon and N. C. Bols (1997). "Methodology for demonstrating and measuring the photocytotoxicity of fluoranthene to fish cells in culture." Toxicol In Vitro **11**(1-2): 107-119.

Schreiber, T., K. Gassmann, C. Götz, U. Hübenthal, M. Moors, G. Krause, H. F. Merk, N.-H. Nguyen, T. S. Scanlan and J. Abel (2010). "Polybrominated diphenyl ethers induce developmental neurotoxicity in a human in vitro model: evidence for endocrine disruption." Environmental health perspectives **118**(4): 572-578.

Schwarzenbach, R. P., P. M. Gschwend and D. M. Imboden (2003). Environmental Organic Chemistry, 2nd Edition. Hoboken, NJ, USA, John Wiley & Sons, Inc.

Seth, R., D. Mackay and J. Muncke (1999). "Estimating the Organic Carbon Partition Coefficient and Its Variability for Hydrophobic Chemicals." Environmental Science & Technology **33**(14): 2390-2394.

Tanneberger, K., M. Knöbel, F. J. M. Busser, T. L. Sinnige, J. L. M. Hermens and K. Schirmer (2013). "Predicting Fish Acute Toxicity Using a Fish Gill Cell Line-Based Toxicity Assay." Environmental Science & Technology **47**(2): 1110-1119.

ter Laak, T. L., J. C. H. van Eijkeren, F. J. M. Busser, H. P. van Leeuwen and J. L. M. Hermens (2009). "Facilitated Transport of Polychlorinated Biphenyls and Polybrominated Diphenyl Ethers by Dissolved Organic Matter." Environmental Science & Technology **43**(5): 1379-1385.

Trapp, S. and R. W. Horobin (2005). "A predictive model for the selective accumulation of chemicals in tumor cells." European Biophysics Journal **34**(7): 959-966.

Yazdanian, M., S. L. Glynn, J. L. Wright and A. Hawi (1998). "Correlating Partitioning and Caco-2 Cell Permeability of Structurally Diverse Small Molecular Weight Compounds." Pharmaceutical Research **15**(9): 1490-1494.

Zaldivar, J. M., M. Mennecozzi, P. Macko, R. Rodrigues, M. Bouhifd and J. Baraibar (2011). A biology-based dynamic approach for the modelling of toxicity in cell assays: Part II: Models for cell population growth and toxicity. Ispra, Italy, European Commission, Joint Research Centre, Institute for Health and Consumer Protection.
